# *Enterobacterales* draft genome sequences: 15 historical (1998–2004) and 30 contemporary (2015–2016) clinical isolates from Pakistan

**DOI:** 10.1128/MRA.00163-23

**Published:** 2023-07-28

**Authors:** Matthew A. Crawford, Christine Lascols, Sara Lomonaco, Ruth E. Timme, Debra J. Fisher, Kevin Anderson, David R. Hodge, Stephen A. Morse, Segaran P. Pillai, Shashi K. Sharma, Erum Khan, Marc W. Allard, Molly A. Hughes

**Affiliations:** 1 Division of Infectious Diseases and International Health, Department of Medicine, University of Virginia, Charlottesville, Virginia, USA; 2 National Center for Emerging and Zoonotic Diseases, Centers for Disease Control and Prevention, Atlanta, Georgia, USA; 3 IHRC, Inc., Atlanta, Georgia, USA; 4 Division of Microbiology, Office of Regulatory Science, Center for Food Safety and Applied Nutrition, US Food and Drug Administration, College Park, Maryland, USA; 5 Science and Technology Directorate, US Department of Homeland Security, Washington, DC, USA; 6 Office of the Commissioner, US Food and Drug Administration, Silver Spring, Maryland, USA; 7 Department of Pathology and Microbiology, Aga Khan University, Karachi, Pakistan; University of Maryland School of Medicine, Baltimore, Maryland, USA

**Keywords:** whole-genome sequencing, antimicrobial resistance, AMRFinderPlus, Pakistan, *Enterobacterales*, *Serratia*, *Salmonella*, *Enterobacter*, *Klebsiella*

## Abstract

The continued emergence and spread of antimicrobial resistance among pathogenic bacteria are ever-growing threats to health and economy. Here, we report the draft genomes for 45 *Enterobacterales* clinical isolates, including historical and contemporary drug-resistant organisms, obtained in Pakistan between 1998 and 2016: 5 *Serratia*, 3 *Salmonella*, 3 *Enterobacter*, and 34 *Klebsiella*.

## ANNOUNCEMENT

*Enterobacterales* is an order of Gram-negative bacteria that includes pathogenic genera responsible for causing life-threatening infections (e.g., *Serratia*, *Salmonella*, *Enterobacter*, and *Klebsiella*) (1). The increasing ability of these organisms to counter antimicrobial therapy represents a serious threat to global health (2, 3). Here, we report the draft genome sequences of 45 clinical isolates of *Enterobacterales* from Pakistan (Table 1), including 5 *Serratia* spp.*,* 3 *Salmonella enterica*, 3 *Enterobacter* spp., and 34 *Klebsiella* spp. These organisms were isolated in 1998 (*n* = 10 strains), 2004 (*n* = 5), or 2015–2016 (*n* = 30) from human urine (*n* = 16), tracheal/respiratory aspirate (*n* = 13), blood (*n* = 9), cerebrospinal fluid (*n* = 3), body fluid/abscess (*n* = 2), stool (*n* = 1), or wounds (*n* = 1). Comparative genomic analyses of *Enterobacterales* will benefit from whole genome sequencing data obtained from bacterial isolates collected at different time points, particularly when considering the evolution of chromosomal-based resistance mechanisms and the transfer of mobile genetic elements that confer the ability to inactivate antibiotics or protect drug targets.

**TABLE 1:** Bacterial isolates, assembly statistics, and accession numbers reported in this study

Isolate^*a*^	Year isolated	CFSAN ID no.^*b*^	Total reads	No. of contigs	N50	Genome coverage	Sequence length	% GC	Assembly accession	Raw-reads accession	No. of AMR genes^*c*^
*Serratia* spp.^*d*^											
BU15418	1998	059609	472,107	37	301,908	50x	5,335,186	59	GCA_014947035.1	SRR12823656	14
BT3288	1998	059619	1,172,352	33	1,038,696	121x	4,908,533	59	GCA_014840855.1	SRR12823812	3
BT2788	2004	059606	3,328,904	29	301,743	318x	5,082,597	59	GCA_014947055.1	SRR12823811	7
BT2790	2004	059607	583,739	27	331,295	62x	5,082,687	59	GCA_014947025.1	SRR12823698	7
BL17834	2015	059633	901,894	31	334,321	93x	5,120,902	60	GCA_014840835.1	SRR12823584	6
*Salmonella enterica*											
BL31130	2015	059647	733,687	69	204,379	72x	4,832,750	52	GCA_014840885.1	SRR12823586	11
BL16732	2016	059648	526,252	70	161,318	52x	4,832,216	52	GCA_014840765.1	SRR12823506	11
BS09	2016	059649	761,351	53	204,375	74x	4,741,340	52	GCA_014840755.1	SRR12823559	1
*Enterobacter* spp*.*^*e*^											
BT2369	1998	059613	922,955	33	500,699	96x	4,699,470	55	GCA_014842835.1	SRR12823534	4
BT2508	1998	059615	774,963	74	307,315	82x	5,145,128	55	GCA_014842795.1	SRR12823808	19
BL36213	2015	059636	1,292,547	136	100,222	132x	5,083,345	55	GCA_014840875.1	SRR12823810	16
*Klebsiella* spp.^*f*^											
BT1645	1998	059610	469,450	47	360,822	49x	5,390,398	57	GCA_014840735.1	SRR12823651	5
BT1995	1998	059611	482,717	45	330,488	39x	5,542,808	57	GCA_014842805.1	SRR12823553	7
BT2390	1998	059612	396,239	107	223,693	41x	5,663,961	57	GCA_014840705.1	SRR12823560	14
BU6451	1998	059614	998,929	37	383,437	105x	5,388,633	58	GCA_014842775.1	SRR12823533	8
BA2858	1998	059616	866,374	63	373,192	90x	5,479,427	57	GCA_014840695.1	SRR12823804	16
BC594	1998	059617	742,867	31	396,025	77x	5,391,043	57	GCA_014840655.1	SRR12823805	5
BT5986	2004	059603	1,137,533	156	169,015	72x	5,470,823	57	GCA_014947005.1	SRR12823585	18
BL8121	2004	059605	813,622	30	475,955	86x	5,359,589	57	GCA_014840775.1	SRR12823799	5
BU10650	2004	059608	474,725	55	280,135	50x	5,391,767	57	GCA_014840795.1	SRR12823669	13
BU66517	2015	059620	904,131	124	279,879	94x	5,597,475	57	GCA_014842715.1	SRR12823666	24
BU67806	2015	059621	1,212,033	126	279,879	125x	5,599,631	57	GCA_014946955.1	SRR12823556	24
BC2272	2015	059622	1,250,440	75	347,090	129x	5,579,117	57	GCA_014840635.1	SRR12823563	20
BU68752	2015	059623	1,442,876	90	300,305	149x	5,630,532	57	GCA_014840605.1	SRR12907791	20
BU20572	2015	059628	878,977	63	270,411	91x	5,514,553	57	GCA_014840575.1	SRR12823564	17
BA4075	2015	059629	667,695	121	215,152	69x	5,773,891	57	GCA_014840535.1	SRR12823697	26
BU17727	2015	059630	611,778	72	253,340	63x	5,755,239	57	GCA_014840505.1	SRR12823696	16
BU2342	2015	059631	1,087,545	69	266,580	110x	5,721,526	57	GCA_014840495.1	SRR12823801	17
BT3680	2015	059632	606,807	80	197,360	62x	5,943,451	57	GCA_014840405.1	SRR12823785	23
BL20957	2015	059634	563,423	85	225,731	58x	5,855,306	57	GCA_014840435.1	SRR12823566	25
BC984	2015	059635	692,167	149	110,045	74x	5,409,046	57	GCA_014840415.1	SRR12823667	20
BL8820	2015	059637	524,961	81	363,896	54x	5,707,288	57	GCA_014946945.1	SRR12823565	14
BL3806	2016	059624	671,026	111	209,522	70x	5,846,639	57	GCA_014840595.1	SRR12907708	24
BU14447	2016	059625	903,753	118	200,842	93x	5,860,281	57	GCA_014946935.1	SRR12907707	19
BT3607	2016	059626	702,416	109	247,654	72x	5,816,877	57	GCA_014840645.1	SRR12823807	29
BU28473	2016	059627	927,936	134	170,964	97x	5,869,562	57	GCA_014840555.1	SRR12823550	26
BU21245	2016	059638	670,239	89	200,608	70x	5,754,316	57	GCA_014840475.1	SRR12823549	25
BT3282	2016	059639	615,831	90	211,172	64x	5,881,145	57	GCA_014840395.1	SRR12907711	19
BT4142	2016	059640	563,603	100	225,002	58x	5,895,020	57	GCA_014840315.1	SRR12823780	23
BU34760	2016	059641	957,114	91	256,824	100x	5,708,320	57	GCA_014946925.1	SRR12823670	15
BL23316	2016	059642	929,840	126	310,596	98x	5,750,634	57	GCA_014840325.1	SRR12823800	20
BU36706	2016	059643	1,261,653	125	270,451	131x	5,870,777	57	GCA_014840345.1	SRR12823781	25
W4192	2016	059644	1,106,558	95	362,791	118x	5,700,681	57	GCA_014840295.1	SRR12823572	22
BU26003	2016	059645	1,301,391	96	190,603	137x	5,647,265	57	GCA_014840305.1	SRR12823567	24
BU26203	2016	059646	531,751	134	147,707	55x	5,864,769	57	GCA_014840245.1	SRR12823587	33

^*a*^ Isolation source: human urine (BU), tracheal/respiratory aspirate (BT), blood (BL), stool (BS), body fluid/abscess (BA), cerebrospinal fluid (BC), or wounds (W).

^*b*^ Isolate identification number assigned by the US Food and Drug Administration (FDA) Center for Food Safety and Applied Nutrition (CFSAN) for sample processing and sequencing.

^*c*^ AMR genes were detected using the NCBI Antimicrobial Resistance Gene Finder tool AMRFinderPlus. Reported counts include complete genes, translated hidden Markov model (HMM) sequences, and point mutations associated with antimicrobial-resistant phenotypes. Full AMR genotypes (including partial and mistranslated AMR genes), as well as stress and virulence genotypes for each clinical isolate, can be accessed using the Pathogen Detection Isolates Browser available at https://www.ncbi.nlm.nih.gov/pathogens/isolates/#

^*d*^ *Serratia marcescens*: BU15418, BT2788, BT2790, and BL17834; *Serratia rubidaea*: BT3288.

^*e*^ *Enterobacter hormaechei*: BT2369 and BT2508; *Enterobacter cloacae*: BL36213.

^*f*^ *Klebsiella variicola*: BT1995; *Klebsiella quasipneumoniae*: BU6451, BU66517, and BU67806; *Klebsiella pneumoniae*: all others.

Isolates were collected using standard methods in clinical microbiology, including growth on differential medium (e.g., MacConkey agar), and maintained as frozen stocks (4). All organisms were de-identified specimens and, thus, exempt from human subjects research requirements as determined by the University of Virginia Institutional Review Board for Health Sciences Research. For DNA isolation, bacteria were grown on lysogeny broth (LB)-Lennox agar and a single colony was subsequently inoculated into LB liquid medium. DNA was extracted using the DNeasy Blood and Tissue Kit (Qiagen). The Nextera XT DNA Library Preparation Kit (Illumina) was used to prepare DNA libraries. Sequencing was performed on an Illumina MiSeq system using MiSeq Reagent Kit v2 (2 × 250 bp paired-end). Reads were assessed for quality (Q > 26) using FastQC v0.11 (5), and taxonomic identity was affirmed with Kraken v1 (6). Shovill v0.9, available in GalaxyTrakr (7), was used to generate *de novo* assemblies; the “trim reads” option was selected and 500 bp was set as the minimum contig length. The NCBI Prokaryotic Genome Annotation Pipeline was used to annotate all draft genomes (8). Except as noted, default parameters were used in all analyses. Genome sequence/assembly statistics are presented in Table 1.

Bacterial identification and phenotypic antimicrobial susceptibility testing were performed using Vitek 2 (bioMérieux). Susceptibility-testing results were confirmed using broth microdilution (9 - 11). Antibiogram data are included in the BioSample record of each strain. Antimicrobial resistance (AMR) genes were identified using AMRFinderPlus v3.11 (12). A total of 117 different AMR genes were found. The most commonly detected AMR genes were *fosA* (91% of all isolates) and *ermD* (89%), which encode a fosfomycin-inactivating glutathione S-transferase and erythromycin resistance methylase, respectively. Other frequently detected AMR genes included those encoding a quinolone efflux pump (*oqxA*; 84%), a sulfonamide-resistant dihydropteroate synthase (*sul1*; 62%), and an extended spectrum beta-lactamase (*bla*_CTX-M-15_; 62%). The average number of AMR determinants detected per organism was 9.7 genes for strains isolated in 1998/2004, increasing to 19.8 genes for those isolated in 2015–2016.

## Data Availability

The genome assemblies described in this report are the first versions and are available in DDBJ/EMBL/GenBank as part of BioProject PRJNA342326 (*Serratia, Salmonella, Enterobacter*) and PRJNA308116 (*Klebsiella*). Assembly and raw-reads accession numbers for each isolate are provided in Table 1 and are hyperlinked to the publicly available data record.
